# Characteristics and risk factors for readmission in HIV-infected patients with *Talaromyces marneffei* infection

**DOI:** 10.1371/journal.pntd.0011622

**Published:** 2023-10-10

**Authors:** Minjuan Shi, Yaqin Qin, Shanshan Chen, Wudi Wei, Sirun Meng, Xiaoyu Chen, Jinmiao Li, Yueqi Li, Rongfeng Chen, Jinming Su, Zongxiang Yuan, Gang Wang, Yingmei Qin, Li Ye, Hao Liang, Zhiman Xie, Junjun Jiang

**Affiliations:** 1 Guangxi Crucial Laboratory of AIDS Prevention and Treatment & School of Public Health, Guangxi Medical University, Nanning, Guangxi, China; 2 The fourth People’s Hospital of Nanning, Nanning, Guangxi, China; 3 Joint Laboratory for Emerging Infectious Diseases in China (Guangxi)-ASEAN, Life Sciences Institute, Guangxi Medical University, Nanning, Guangxi, China; OSWALDO CRUZ FOUNDATION, BRAZIL

## Abstract

**Objectives:**

*Talaromyces marneffei* (*T*. *marneffei*) is an opportunistic fungal infection (talaromycosis), which is common in subtropical regions and is a leading cause of death in HIV-1-infected patients. This study aimed to determine the characteristics and risk factors associated with hospital readmissions in HIV patients with *T*. *marneffei* infection in order to reduce readmissions.

**Methods:**

We conducted a retrospective study of admitted HIV-infected individuals at the Fourth People’s Hospital of Nanning, Guangxi, China, from 2012 to 2019. Kaplan-Meier analyses and Principal component analysis (PCA) were used to evaluate the effects of *T*. *marneffei* infection on patient readmissions. Additionally, univariate and multifactorial analyses, as well as Propensity score matching (PSM) were used to analyze the factors associated with patient readmissions.

**Results:**

HIV/AIDS patients with *T*. *marneffei*-infected had shorter intervals between admissions and longer lengths of stay than non-*T*. *marneffei*-infected patients, despite lower readmission rates. Compared with non-*T. marneffei*-infected patients, the mortality rate for talaromycosis patients was higher at the first admission. Among HIV/AIDS patients with opportunistic infections, the mortality rate was highest for *T*. *marneffei* at 16.2%, followed by cryptococcus at 12.5%. However, the readmission rate was highest for cryptococcus infection (37.5%) and lowest for *T*. *marneffei* (10.8%). PSM and Logistic regression analysis identified leukopenia and elevated low-density lipoprotein (LDL) as key factors in *T.marneffei*-infected patients hospital readmissions.

**Conclusions:**

The first admission represents a critical window to intervene in the prognosis of patients with *T*. *marneffei* infection. Leukopenia and elevated LDL may be potential risk factors impacting readmissions. Our findings provide scientific evidence to improve the long-term outcomes of HIV patients with *T*. *marneffei* infection.

## Author summary

Talaromyces marneffei is an opportunistic fungal infection primarily affecting individuals with impaired immune function, namely those with HIV-infected. Previous studies from Guangxi, China have reported approximately 17% mortality among HIV/AIDS patients with talaromycosis. Our study reveals a 10.8% readmission rate and a 16.2% mortality rate among HIV/AIDS patients who were exclusively infected with *T*. *marneffei* during the first admission. Understanding these key prognostic factors and the timing of intervention are crucial for clinicians to improve patient outcomes and reduce hospital readmissions. To address this issue, we conducted an extensive 8-year retrospective study involving 12,946 HIV/AIDS patients. Compared to the eight major common opportunistic infections, *T. marneffei*-infected patients had the lowest readmission rate, yet accounted for the highest mortality rate. Leucopenia and elevated LDL emerged as significant risks for increasing hospital readmissions. Importantly, our study highlights the critical window for intervening in talaromycosis prognosis–the first hospitalization. These findings have major clinical implications, enabling clinicians to implement effective treatments and interventions starting at first hospital admission to improve prognosis in HIV/AIDS patients with *T*. *marneffei*-infected.

## Introduction

Talaromyces marneffei (*T*. *marneffei*, formerly known as *Penicillium marneffei*) is the causative of talaromycosis, a fatal systemic fungal infection. Talaromycosis has become widespread throughout Southeast Asian countries (Thailand, Vietnam, Malaysia, India) and southern China, including the Pearl River Basin (Guangxi, Guangdong, Yunnan, Hong Kong, Taiwan) [1–3]. Cases of talaromycosis in non-endemic regions are typically travel-related and linked to visits to endemic areas, additionally, the annual increase in migration from these regions has been attributed to exported cases [4,5]. In Southeast Asia, *T*. *marneffei* is responsible for approximately 50,000 new infections and up to 5000 deaths annually [6,7]. Guangxi, China has a high HIV/AIDS burden and the third highest cumulative cases count. Hospitalized HIV-infected individuals have a high *T*. *marneffei* infection rate (up to 16.1%), and a higher mortality rate than most common HIV-related complications (AHR = 4.52) [8–10]. *T*. *marneffei* infection often causes delayed or incorrect diagnosis due to non-specific symptoms, allowing for disease progression in these individuals. Delayed antifungal therapy reduces patients’ quality of life and increases healthcare costs for these patients [11,12].

*T*. *marneffei* infection is often associated with variable symptoms which can include severe tissue damage and sepsis [13]. This can worsen disease, increase mortality, and lead to hospital readmissions in people with HIV/AIDS. Frequent hospitalizations impose financial burdens on families, strain the healthcare system, and increase complications and opportunistic infection risks. For example, readmissions for sepsis increase skin and respiratory infection risks [14]. Reducing hospital readmissions for diabetes and heart failure can improve quality of life and decrease opportunistic infection risks [15,16]. While *T*. *marneffei’s* epidemiology and treatment are well-studied, research on readmission characteristics and risk factors of patients readmitted to the hospital for *T*. *marneffei* infection is limited. Further investigation is necessary to identify the readmissions characteristics and risk factors associated with *T*. *marneffei*-infected patients, and the adverse effects of different types of opportunistic infections causing hospitalization.

We aimed to assess the clinical characteristics and risk factors contributing to hospital readmissions of HIV/AIDS patients presenting with talaromycosis. We analyzed laboratory test results and treatment outcomes from four consecutive hospitalizations to identify differences and characteristics among patients. Our findings provide valuable insights for clinicians treating HIV/AIDS patients with talaromycosis, which could potentially improve clinical treatment prognosis.

## Method

### Ethics statement

This study was approved by the Human Research Ethics Committee of Guangxi Medical University (Ethical Review No. 20210099)

### Study population and data collection

This comprehensive retrospective cohort study was conducted at the Fourth People’s Hospital of Nanning, the primary tertiary healthcare facility for infectious diseases in Guangxi and the region’s largest HIV/AIDS treatment center. The study included 12,946 HIV/AIDS patients admitted to the medical center between January 2012 and June 2019. We retrospectively tracked data from four consecutive admissions of patients with continuous readmissions. Data were obtained from the hospital’s electronic medical record system, including diagnoses, laboratory test results, and outcomes during hospitalization, and underwent manual calibration and review. Each admission was divided into two groups based on the presence of *T*. *marneffei* infection. Patients were further separated into two groups based on whether or not readmitted to the hospital. Demographic information, ART status, biochemical, routine blood results, CD3/4/8 T-cell counts, and discharge complications diagnosed were collected. Inclusion criteria included 1) HIV infection confirmed by positive Elisa and western blotting. (2) Talaromycosis diagnosis based on *T*. *marneffei* was isolated /cultured from blood, skin tissue, bone marrow, lymph nodes, or other bodily fluid samples, indicating compliance with the diagnostic criteria.

### Definitions of various complications and coinfections

The focus of this study was hospital readmission, which was categorized as either “No” for no further admission or “Yes” for readmissions, based on subsequent readmission records. Leukopenia was defined as a white blood cell (WBC) count < 3.5×10^9^/L, while Leukocytosis was defined as a WBC count > 9.5×10^9^/L. Diagnosis of pneumonia included bacterial pneumonia, viral pneumonia, pulmonary mycosis (including pneumocystis pneumonia), and other pneumonia, but excluded pulmonary tuberculosis pneumonia, which was classified as tuberculosis [17]. The definition of meningitis includes purulent meningitis, cryptococcosis meningitis, and viral meningitis but does not include tuberculosis meningitis, which is classified as tuberculosis [18]. Chronic hepatitis B/C and candidiasis were confirmed by standard infectious disease diagnostic criteria [19]. Residual complications and coinfections were defined according to standards of Internal Medicine guidelines [20].

### Statistical analysis

All analyses were conducted using the Statistical Package for the Social Sciences version 26.0 (SPSS Inc, Chicago, IL, USA) and R Studio (4.0.6). Results are presented as numbers, frequencies (%), or medians with interquartile ranges (IQR). ​The chi-square test and Mann–Whitney U test were applied to categorical variables and nonnormally distributed data, respectively. Kaplan–Meier analysis was used to calculate the mortality rates, and the significance was assessed using log-rank tests. Logistic regression analysis utilized a forward selection method for variable inclusion in the model, and 1:1 PSM was used to match the confounding factors. Principal component analysis (PCA) evaluated the effects of *T*. *marneffei* infection on patient readmissions. A two-sided *p*-value <0.05 was considered statistically significant.

## Result

### General characteristics of study participants

This study investigated the demographic characteristics of patients with different types of infections (S1 Table). The age distribution was primarily 20–60 years, interestingly 45% of herpesvirus-infected patients were over 60 years old. Most patients were male (70%) and of Han ethnicity (57%). Over 58% of patients were married, while the proportion of single and married individuals was relatively equal (around 48%) among those with Treponema pallidum (*T*. *pallidum*) infection. Farmers accounted for 40% of patients with *T*. *pallidum* infection. Antiviral treatment was uncommon across all infection types. These findings demonstrate the need for targeted prevention strategies accounting for patient demographics.

### Readmission rates among HIV-infected patients with different types of opportunistic infections

**Fig 1:**
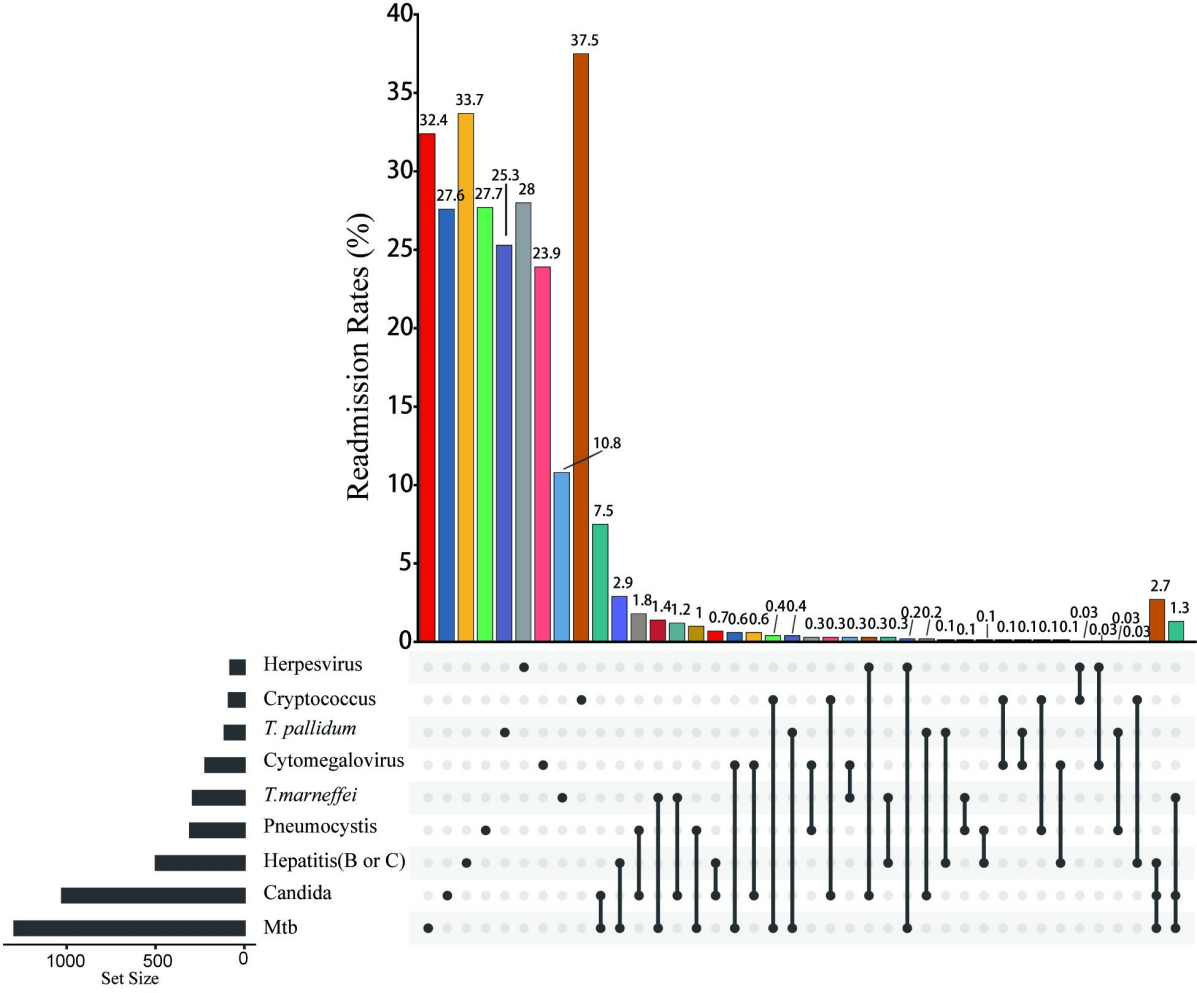
Analysis of hospital readmission rates among HIV/AIDS patients with different types of opportunistic infections. **Abbreviations:** Mtb, mycobacterium tuberculosis; *T*. *pallidum*, Treponema pallidum.

Previous studies have documented the prevalence of three key leading pathogens associated with opportunistic infections (namely, Cryptococcus, Mycobacterium tuberculosis (Mtb), and *T*. *marneffei*) in individuals with HIV infections in Vietnam. Nevertheless, follow-up on the hospital readmissions associated with these infections has not been reported [21–23]. This study analyzed the readmission rates of HIV-infected patients with different types of opportunistic infections. Our findings revealed that cryptococcus infection had the highest hospital readmission rate at 37.5%, closely followed by hepatitis (B or C) infection at 33.7%, Mtb infection had a rate of 32.4%, however *T*. *marneffei* infection with the lowest rate at 10.8%. Further analysis of combined infections caused by nine viruses showed the following: in dual coinfections, the highest incidence was observed with Mtb and Candida coinfection at 7.5%; in triple coinfections, the highest incidence was found with Mtb, Candida, and hepatitis (B or C) coinfection at 2.7% (Fig 1).

### Mortality rates among HIV-infected patients with different types of opportunistic infections

**Fig 2:**
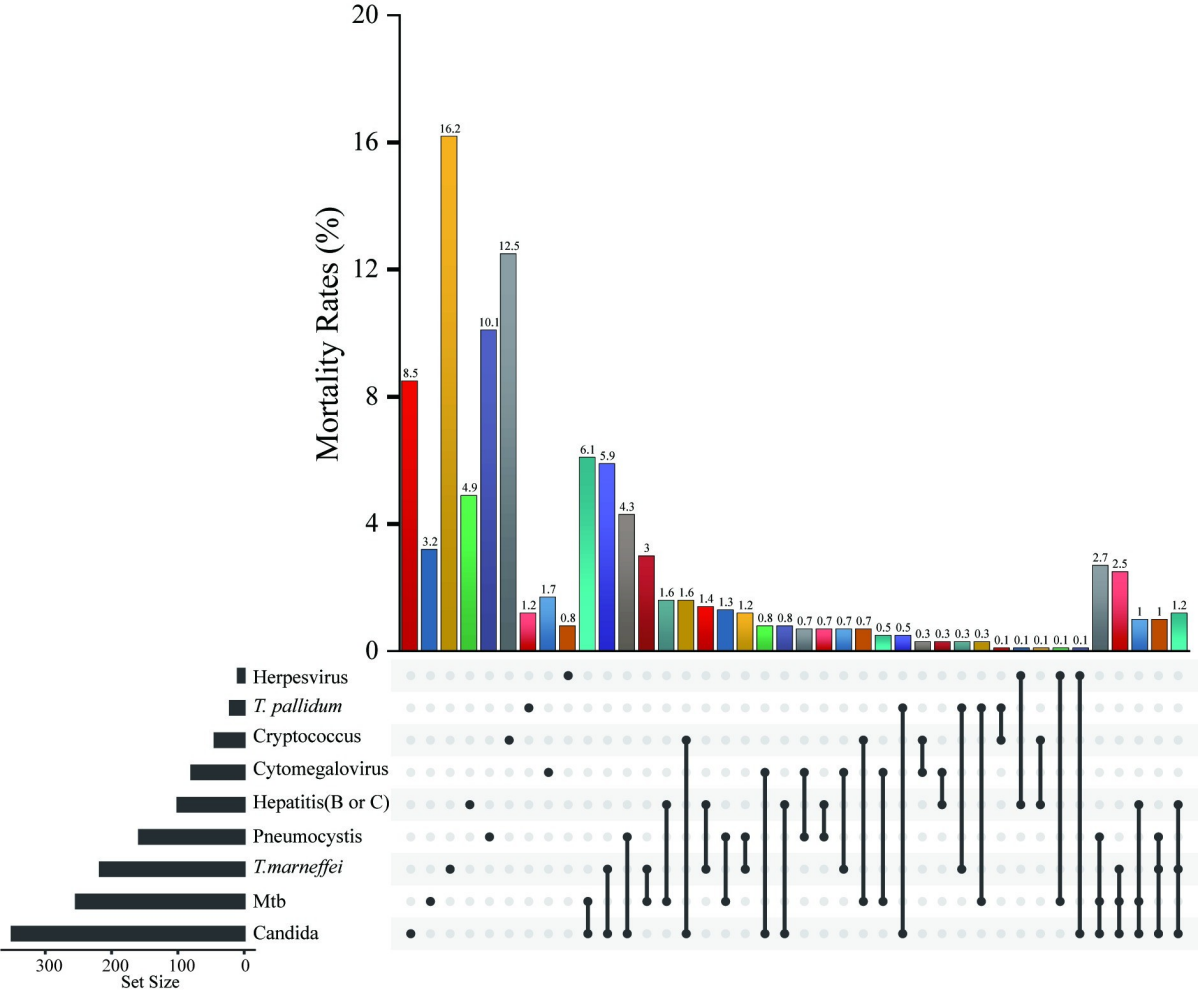
Analysis of mortality rates among HIV/AIDS patients with different types of opportunistic infections. **Abbreviations:** Mtb, mycobacterium tuberculosis; *T*. *pallidum*, Treponema pallidum.

Previous studies indicate opportunistic infections significantly impact HIV/AIDS patient survival outcomes [24–26]. However, the effect of different pathogenic infections and coinfection numbers on outcomes in *T*. *marneffei*-infected patients remains unclear. To address these gaps in the field, we evaluated the mortality rates for patients with different types of opportunistic infections and compared *T*. *marneffei*-infected patients’ mortality rates with a variety of different coinfections. Interestingly, *T*. *marneffei* infection had the highest mortality rate at 16.2%, followed by cryptococcus infection at 12.5%, pneumocystis infection at 10.1%, Candida infection at 8.5%, and herpesvirus infection with the lowest rate of 0.8%. Surprisingly, the mortality rates of talaromycosis patients who presented with other opportunistic infections were lower than those of patients with only *T*. *marneffei* infection. For *T*. *marneffei* and Mtb dual coinfection, the mortality rate was 3.0%. For triple coinfection with *T*. *marneffei*, Mtb, and Candida, the mortality rate for the composite infection was 2.5% (Fig 2).

### PCA of the effect of opportunistic infections

**Fig 3:**
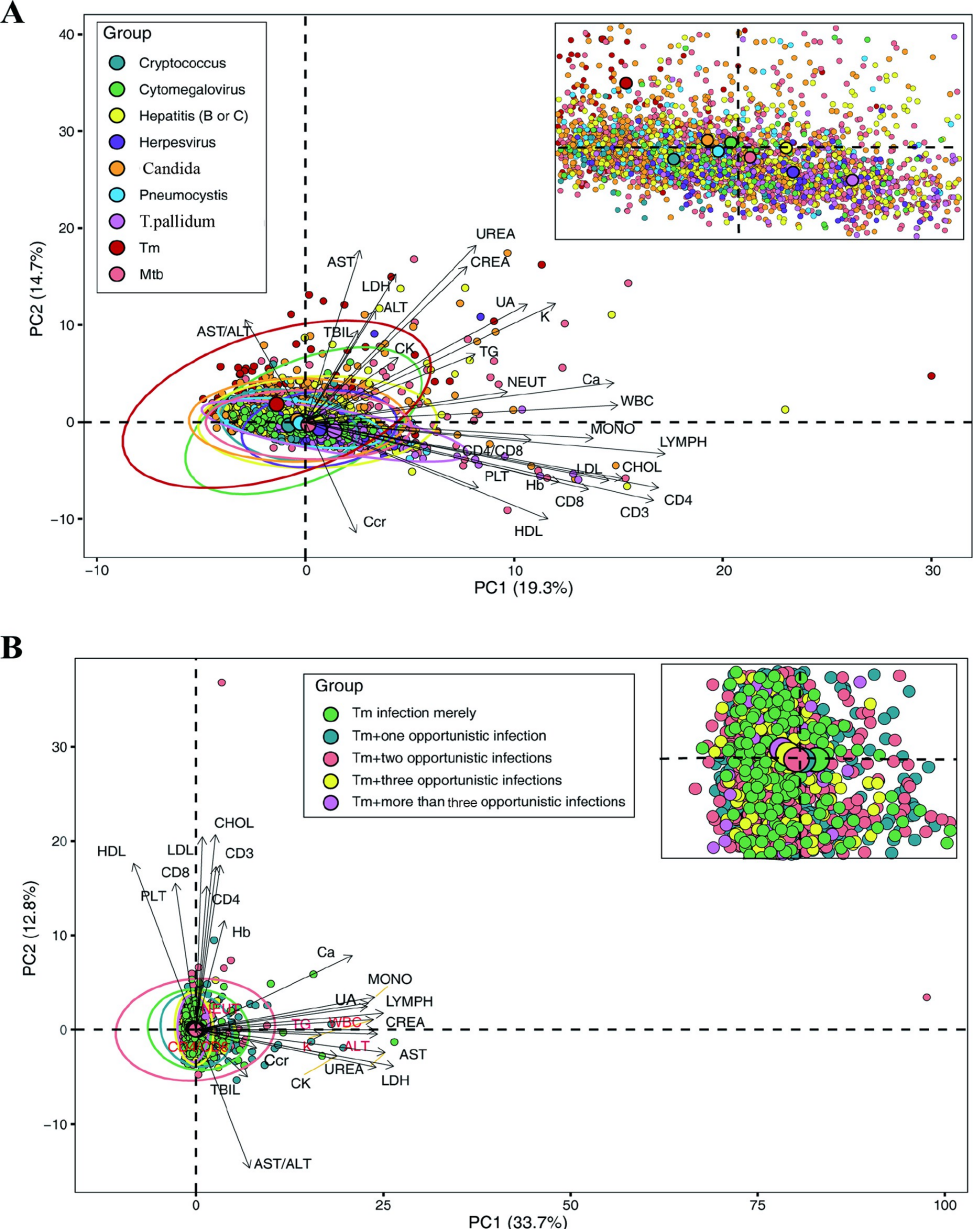
PCA among HIV-infected patients with different types of opportunistic infections. (A). PCA among HIV patients with different types of opportunistic infections. (B). PCA among Talaromycosis patients with different opportunistic coinfections. Tm, *T*. *marneffei*; Mtb, mycobacterium tuberculosis; T. pallidum, Treponema pallidum.

The above findings showed that HIV-infected patients with *T*. *marneffei* had the lowest hospital readmission rate but the highest mortality rate compared to other combinations of opportunistic infections. This raised our concern, as *T*. *marneffei* tends to immunocompromised HIV/AIDS patients. To further investigate the immune status of different types of opportunistic infections, Principal component analysis (PCA) was performed on the laboratory findings (Fig 3A).

For patients with a single opportunistic infection, loading coefficients (length of the projection of variables on the PC1 axis), in general, showed variables closely related to PC1, with LYMPH, CD4, and CD3 having a strong influence on PC1 (quality correlation >0.6). These were projected to the right of the 0 point, indicating a positive relationship with PC1, which mainly responded to immune status. Similarly, PC2 responded mainly to heart, liver, and kidney organ function. The most influential variables were UREA, AST, CREA, and LDH in descending order.

The center point position of the patient distribution on the PC1 axis represented the patient’s group value on PC1. A higher value means a higher PC1 score, with positive and negative relationships on both sides of the 0 point. Talaromycosis patients were positioned to the left of the 0 point on the PC1, with the largest projection, suggesting a negative immune correlation of *T*. *marneffei* infection in HIV/AIDS patients. Cryptococcus exhibited the second-highest mortality rate. Surprisingly, talaromycosis patients had relatively mild organ infections. Analysis of talaromycosis patients with different coinfection numbers showed that although PC1 axis position was ordered by the number of coinfections, it did not effectively distinguish the groups (Fig 3B).

### Analysis of readmissions among *T*. *marneffei*-infected and non-*T*. *marneffei*-infected patients

To gain deeper insights into hospital readmission patterns within the talaromycosis population, a comprehensive investigation was conducted encompassing four consecutive admissions, categorized into discrete cohorts based on the presence or absence of *T*. *marneffei* infections. After the first admission, an upward trajectory was discerned in the frequency of second, third, and fourth readmission for the *T. marneffei*-infected group, with rates of 16.5%, 18.7%, and 35.2%, respectively (χ^2^_trend_ = 8.609, *p* = 0.003). However, it was imperative to acknowledge that these proportions remained comparatively lower than the non-*T. marneffei*-infected group, which registered corresponding rates of 29.1%, 38.3%, and 48.4%. Significant differences in the second and third readmission rates were observed between the two groups (*p*<0.001), while the fourth readmission rate did not reach statistical significance (*p* = 0.057). The talaromycosis population exhibited a lower percentage of readmissions, however, the interval between hospital admissions was shorter, with an average reduction of 25 days, and the length of hospital stay was longer, with an average increase of 6.5 days longer on average (Table 1).

**Table 1:** Analysis of hospitalization among HIV infection without/with *T*. *marneffei* infection at 1–4 admissions.

		1			2			3			4		

Admission number		Without Tm infection (n = 11205)	With Tm infection (n = 1741)	*p*	Without Tm infection (n = 3262)	With Tm infection (n = 288)	*p*	Without Tm infection (n = 1250)	With Tm infection (n = 54)	*p*	Without Tm infection (n = 3262)	With Tm infection (n = 19)	*P*
Percentage of readmissions		-	-		29.1% (3262/11205)	16.5% (288/1741)	<0.001	38.3% (1250/3263)	18.7% (54/289)	<0.001	48.4% (605/1250)	35.2% (19/54)	0.057
Time between hospital readmissions(days)#		-	-		86 (39,325)	59 (36,100)	<0.001	82 (35,260)	49 (36,114)	0.023	73 (29,238)	58 (32,86)	0.187
Time of hospitalization(days)#		14 (7,22)	21 (13,30)	<0.001	12 (6,20)	18 (9,28)	<0.001	11 (6,19)	16 (8,24)	0.003	10 (6,18)	18 (10,28)	0.041
Prognosis (%)	Cured, improved	9959 (88.0)	1449 (83.2)		3006 (92.2)	255 (88.5)		1136 (90.9)	51 (94.4)		548 (90.6)	16 (84.2)	
	Untreated, ineffective	332 (3.0)	54 (3.1)		44 (1.3)	6 (2.1)		16 (1.3)	0 (0.0)		15 (2.5)	0 (0.0)	
	Death	550 (4.9)	217 (12.5)	<0.001	156 (4.8)	20 (6.9)	0.207	70 (5.6)	1 (1.9)	0.462*	29 (4.8)	2 (10.5)	0.271
	Other	364 (3.2)	21 (1.2)		56 (1.7)	7 (2.4)		28 (2.2)	2 (3.7)		13 (2.1)	1 (5.3)	

# Data are presented as the median [interquartile range (IQR)]

**Fig 4:**
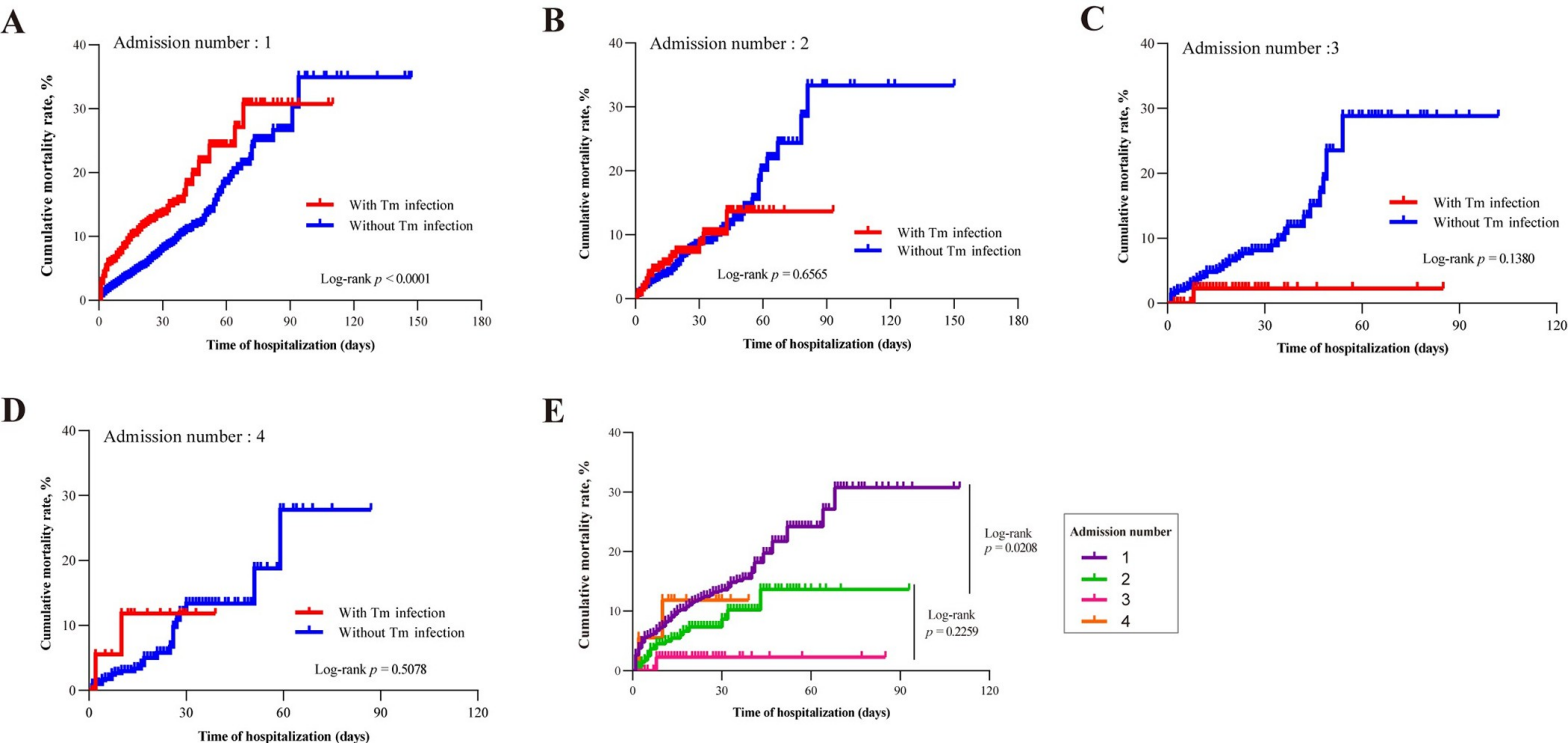
Kaplan–Meier analysis of cumulative mortality among HIV/AIDS patients with and without *T*. *marneffei* infection. (A). First admission to hospital. (B). Second admission to hospital. (C). Third admission to hospital. (D). Fourth admission to hospital. (E). Survival curve for the first to fourth hospital admission in HIV/AIDS patients with *T*. *marneffei* infection. Statistical significance was measured by the log-rank test.

In terms of prognosis, we focused more on the cured/improved and mortality rates. The mortality rate within 30 days of the first admission was 11.3% among talaromycosis patients, exceeding the 4.3% mortality among patients without *T*. *marneffei* infection. The rates of cure/improvement were lower in the first admission of talaromycosis patients compared to those with non-*T*. *marneffei* infections (*p*<0.05), but there was no statistically significant difference between the second, third, and fourth admissions (*p*>0.05). The survival analyses also showed that the cumulative mortality in the *T*. *marneffei*-infected patients was higher than in *T*. *marneffei*-uninfected patients at first admission (Fig 4A). In contrast, at the three subsequent follow-up readmissions, there was no statistically significant difference in mortality between the two groups of patients (*p*>0.05) (Fig 4B–4D). Overall, the prognosis difference between *T. marneffei*-infected patients and non-*T. marneffei*-infected patients mainly occurred during the first admission. *T. marneffei*-infected patients had a lower cured/improved rate and higher cumulative mortality rate. For *T. marneffei*-infected patients themselves, the cumulative mortality rate from the first to the third admission seemed to show a decreasing trend (Fig 4E), suggesting that the initial admission was pivotal in affecting the prognosis of the patients of *T*. *marneffei*-infected patients.

### Analysis of factors influencing readmissions

In the results above, we identified that initial hospital admission of the talaromycosis population was a critical time point. Subsequently, we concentrated on the risk factors influencing readmission among the talaromycosis population according to the clinical characteristics of the first admission, including disease spectrum (S2 Table) and laboratory blood test results (S3 Table), and separately included the variables with *p*<0.05 for the first admission in Tables 2 and 3, relatively. Given the limited number of the fourth admission, our subsequent analysis focused on the first and three readmissions.

**Table 2:** Disease spectrum among HIV/AIDS patients with *T*. *marneffei* infection at the first hospital admission.

Complications		No admission (n = 1453)	Admission (n = 288)	*p*
Mycobacterium tuberculosis infection	No	896 (61.7)	134 (46.5)	<0.001
	Yes	557 (38.3)	154 (53.5)	
Pneumocystis carinii infection	No	341 (23.5)	85 (29.5)	0.029
	Yes	1112 (76.5)	203 (70.5)	
Pneumocystis infection	No	1234 (84.9)	258 (89.6)	0.039
	Yes	219 (15.1)	30 (10.4)	
IRIS	No	1442 (99.2)	281 (97.6)	0.019
	Yes	11 (0.8)	7 (2.4)	
Septic shock	No	1363 (93.8)	287 (99.7)	<0.001
	Yes	90 (6.2)	1 (0.3)	

**Table 3:** Laboratory test results among HIV/AIDS patients with *T*. *marneffei* infection for the first hospital admission.

Complications	No admission(n = 1453)	Admission (n = 288)	*p*
LDL (mmol/L)			0.042
≤3.37	1063 (95.7)	206 (92.4)	
>3.37	48 (4.3)	17 (7.6)	
WBC (109/L)			0.022
3.5–9.5	752 (54.8)	137 (48.9)	
<3.5	498 (36.2)	125 (44.7)	
>9.5	124 (9.0)	18 (6.4)	
NEUT (109/L)			0.04
40–75	913 (66.4)	188 (67.1)	
<40	265 (19.3)	66 (23.6)	
>75	196 (14.3)	26 (9.3)	

The result showed that the disease spectrum we collected showed significant differences only at the first admission. Specifically, among those patients readmitted for a second time, a higher prevalence of Mtb infection and IRIS was observed compared to those who were not readmitted, whereas pneumocystis infection, pneumonia, and septic shock had a lower infection rate (Table 2). In the subsequent two admissions, a balanced distribution was observed between the readmission and non-readmission groups (S2 Table).

These findings once again emphasized the importance of timely intervention during the first admission impacting the prognosis of the talaromycosis population, which can also be reflected in laboratory test results (S3 Table). A greater proportion of those readmitted had elevated LDL than those in the no longer admitted group and a greater percentage of leukopenia, as did decreased neutrophils (Table 3). And the laboratory results of non-*T*. *marneffei* infection could be found in S5 Table. Building on the existing results, we proceed to investigate the factors influencing readmissions following the first hospitalization among talaromycosis patients. Furthermore, the disease spectrum and the laboratory test results for non-*T*. *marneffei* infection can be found in S4 and S5 Tables, relatively, for interested readers.

**Fig 5:**
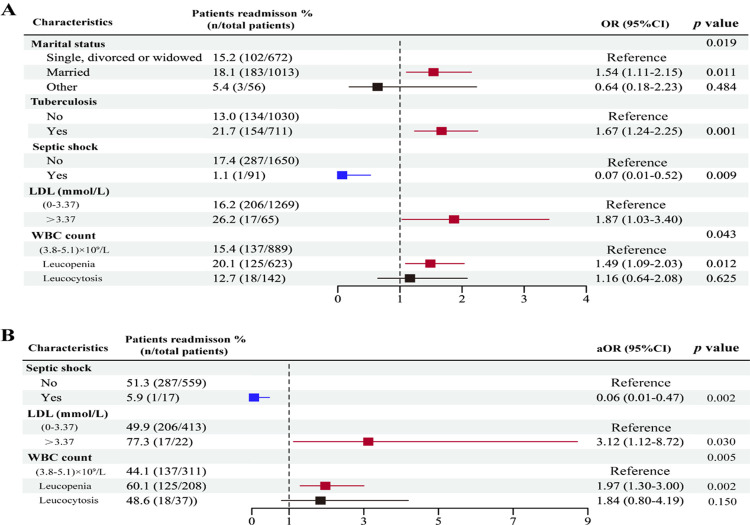
Forest plot showing results of logistics analysis on factors influencing hospital readmission of talaromycosis patients for the first admission. (A). No variables were adjusted, model χ^2^ = 57.921, *p*<0.001; Hosmer–Lemeshow test χ^2^ = 2.268, *p* = 0.994. (B). Adjusted for demography, ART, and opportunistic infections, including Mtb, Candida, pneumocystis, hepatitis (B or C), herpesvirus, cryptococcus, *T*. *pallidum*, cytomegalovirus, model χ^2^ = 29.994, *p*<0.001; Hosmer–Lemeshow test χ^2^ <0.001, *p* = 1.000.

Binary regression equations typically utilize categorical variables to elucidate the positive or negative effects of different assigned variables on the dependent variable concerning the reference level. In our study, we systematically categorized the laboratory test data based on the reference ranges of test results provided by the hospital (S3 Table). To identify the influential factors that contribute to readmission in the talaromycosis population, we utilized demographic information, ART status, complications, and categorical laboratory test results as the independent variables with *p*<0.05. “Readmission” served as the dependent variable in an unconditional logistic regression analysis, the results were presented as a forest plot (Fig 5). The statistical analysis of the model yielded a significant result, and the model fit was satisfactory (all *p*>0.05).

The results showed that several risk factors for readmission during the first admission were identified, including marital status, tuberculosis infection, elevated LDL, and leukopenia (Fig 5A). To control for confounding factors, we adjusted demographics, ART status, and opportunistic infections using a 1:1 propensity score match, and the distribution of these variables between groups were balanced after matching (S6 Table). Fig 5B showed that elevated LDL and leukopenia continued to be significant risk factors for readmissions after matching. Additionally, septic shock consistently demonstrated a negative effect on readmission.

## Discussion

This study initially observed that among eight other opportunistic infections, the talaromycosis population had the lowest readmission rate yet the highest mortality rate. Subsequent PCA indicated that the talaromycosis population exhibit the most compromised immune status, which could plausibly explain the observed high mortality rate. An analysis of consecutive hospital readmissions in populations infected with *T*. *marneffei* revealed that the first admission could represent a crucial window for an intervening point in patient prognosis.

The high elevated prevalence of *T*. *marneffei* infection among patients with poor immune status coupled with the lack of antiviral therapy for 77%, likely contributes to the observation that the talaromycosis population has the poorest immune status. In Table 1 we found that the readmission rate of HIV/AIDS patients infected with *T*. *marneffei* is not higher than that of patients without infection, but in Table 1 and Fig 4, we find that the highest mortality rate among patients infected with *T*. *marneffei* occurs during their first admission, due to the fact that many of the first-time admission are already very ill at the time of readmissions. Whereas *T*. *marneffei* is currently sensitive to both amphoteric penicillins, aggressive antimicrobial therapy can greatly reduce the mortality rate of subsequent *T*. *marneffei* readmissions, as long as the benefits of the medication outweigh the risks at the time of the first admission. This also re-emphasizes the importance of early diagnosis and treatment for the prognosis of HIV/AIDS patients infected with *T*. *marneffei*, which is seizing the golden time point of intervention at first admission to improve patient prognosis.

Our results revealed that readmitted patients with *T*. *marneffei* had a significantly higher prevalence of infections with Mtb, cytomegalovirus, and Candida than non-*T. marneffei*-infected group. Furthermore, the laboratory findings revealed that *T. marneffei*-infected patients exhibited more severe immune cell damage when compared to non-infected patients, as evidenced by the higher percentage of CD4 <200 (cells/ul) (92.7 vs 50.7) and CD8 <190 (cells/ul) (40.6 vs 8.1), which supported by PCA results. It is noteworthy that low CD4 counts have been reported to be associated with an increased risk of severe illness or mortality in patients [27–29]. Talaromycosis is prone to misdiagnosis due to its resemblance to tuberculosis [30]. Therefore, clinicians could perform early pathogenetic testing, providing early intervention and comprehensive care during the first hospitalization, which helps to control infections promptly and reduce the incidence of complications. Previous studies have reported that regular follow-up after discharge, enhanced surveillance, and early outpatient intervention when problems arise can reduce the hospital readmission rate of AIDS patients [31,32].

After correcting for demographics and the eight most common opportunistic infections, sceptic shock, elevated LDL and leucopenia remained as influential factors for hospital readmissions in the talaromycosis population. Leukopenia is associated with a variety of infections [33–35]. For HIV/AIDS patients with talaromycosis who already have compromised immune systems, leukopenia can significantly increase the risk of infections and hospital readmissions. It was necessary to determine the specific type of cell abnormality when patients with an abnormal white blood cell count, which could enable a more accurate determination of its cause. A study in Taiwan also showed an increased risk of stroke in HIV-infected patients, especially those with talaromycosis, which can also lead to hospital admissions [36]. In conclusion, for high-risk patients, clinical physicians can effectively control these two indicators by considering delaying the discharge time, and scheduling early follow-up. It is worth noting that Mtb infection was also a risk factor for hospital readmissions, without considering the interaction between opportunistic infections. Mtb infection typically follows a chronic clinical course, and patients are susceptible to hospital readmissions. Frequent unplanned hospital readmissions in TB patients increase costs and burden, decrease quality of life, which are associated with poorer prognosis [37]. Clinicians could closely monitor high-risk patients with concurrent Mtb and *T*. *marneffei co*infections, and provide proactive treatment for Mtb infection and antiviral therapy.

This study has some limitations that should be noted. We did not collect information on the time between *T*. *marneffei* infection onset and diagnosis, treatment regimens and additional clinical characteristics, antiretroviral regimens, and outcome monitoring. The causal relationship between *T*. *marneffei* infection and immune status could not be determined due to the limitations of the study design.

In summary, the study identifies the critical time for hospital readmissions in the talaromycosis population as the first admission, and the factors that affect hospital readmissions including leukopenia and elevated LDL. This study helps guide precise clinical interventions to improve the prognosis of talaromycosis patients and reduce the healthcare burden.

## Supporting information

S1 Table Characteristics of HIV patients coinfected with different opportunistic infections. (DOCX)

S2 Table Disease spectrum among HIV/AIDS patients with *T*. *marneffei* infection during three consecutive hospital admissions. (DOCX)

S3 Table Laboratory test results among HIV/AIDS patients with *T*. *marneffei* infection during three consecutive hospital admissions. (DOCX)

S4 Table Disease spectrum among HIV/AIDS patients with non-*T*. *marneffei* infection during three consecutive hospital admissions. (DOCX)

S5 Table Laboratory test results among HIV/AIDS patients with non-*T*. *marneffei* infection during three consecutive hospital admissions. (DOCX)

S6 Table Demographic information and ART status among the *T. marneffei* infection population at first admission before and after 1:1 propensity score matching. (DOCX)
