# Supplementary material for: Characteristics and risk factors for readmission in HIV-infected patients with Talaromyces marneffei infection
Source: PLoS Negl Trop Dis. 2023 Oct 10;17(10):e0011622. doi: 10.1371/journal.pntd.0011622 (PMC10564132; doi:10.1371/journal.pntd.0011622)
Supplement: S1 Table — (DOCX) [file pntd.0011622.s001.docx]

**S1 Table. Characteristics among HIV patients coinfected with different opportunistic infections**

| Variable  (n, %) | Tm  infection  (n=1741) | Mtb infection (n=4152) | Candida (n=3911) | Pneumocystis  infection (n=1306) | Hepatitis (B or C) (n=1601) | Herpesvirus  infection (n=287) | Cryptococcus  infection (n=251) | *T.pallidum* infection (n=455) | Cytomegalovirus  infection  (n=1028) |
| --- | --- | --- | --- | --- | --- | --- | --- | --- | --- |
| Age |  |  |  |  |  |  |  |  |  |
| <20 | 40 (2.3) | 76 (1.8) | 52 (1.3) | 24 (1.8) | 12 (0.7) | 2 (0.7) | 2 (0.8) | 4 (0.9) | 11 (1.1) |
| 20-40 | 789 (45.3) | 1325 (31.9) | 1186 (30.3) | 376 (28.8) | 682 (42.6) | 42 (14.6) | 98 (39.0) | 212 (46.6) | 322 (31.3) |
| 41-60 | 679 (39.0) | 1668 (40.2) | 1625 (41.5) | 576 (44.1) | 686 (42.8) | 114 (39.7) | 115 (45.8) | 160 (35.2) | 456 (44.4) |
| >60 | 233 (13.4) | 1083 (26.1) | 1048 (26.8) | 330 (25.3) | 221 (13.8) | 129 (44.9) | 36 (14.3) | 79 (17.4) | 239 (23.2) |
| Sex |  |  |  |  |  |  |  |  |  |
| Male | 1435 (82.4) | 3353 (80.8) | 3044 (77.8) | 994 (76.1) | 1308 (81.7) | 211 (73.5) | 177 (70.5) | 358 (78.7) | 826 (80.4) |
| Female | 306 (17.6) | 799 (19.2) | 867 (22.2) | 312 (23.9) | 293 (18.3) | 76 (26.5) | 74 (29.5) | 97 (21.3) | 202 (19.6) |
| Nationality |  |  |  |  |  |  |  |  |  |
| Han | 1042 (59.9) | 2528 (60.9) | 2295 (58.7) | 753 (57.7) | 1009 (63.0) | 169 (58.9) | 156 (62.2) | 290 (63.7) | 592 (57.6) |
| Zhuang | 656 (37.7) | 1521 (36.6) | 1522 (38.9) | 508 (38.9) | 555 (34.7) | 115 (40.1) | 88 (35.1) | 149 (32.7) | 408 (39.7) |
| Other | 43 (2.5) | 103 (2.5) | 94 (2.4) | 45 (3.4) | 37 (2.3) | 3 (1.0) | 7 (2.8) | 16 (3.5) | 28 (2.7) |
| Marital status |  |  |  |  |  |  |  |  |  |
| Single, divorced or widowed | 672 (38.6) | 1418 (34.2) | 1365 (34.9) | 480 (36.8) | 574 (35.9) | 94 (32.8) | 79 (31.5) | 217 (47.7) | 350 (34.0) |
| Married | 1013 (58.2) | 2602 (62.7) | 2420 (61.9) | 793 (60.7) | 973 (60.8) | 185 (64.5) | 163 (64.9) | 226 (49.7) | 651 (63.3) |
| Other | 56 (3.2) | 132 (3.2) | 126 (3.2) | 33 (2.5) | 54 (3.4) | 8 (2.8) | 9 (3.6) | 12 (2.6) | 27 (2.6) |
| Occupation |  |  |  |  |  |  |  |  |  |
| Farmer | 945 (54.3) | 2348 (56.6) | 2236 (57.2) | 702 (53.8) | 820 (51.2) | 174 (60.6) | 140 (55.8) | 182 (40.0) | 544 (52.9) |
| Unemployed, retired | 356 (20.4) | 941 (22.7) | 886 (22.7) | 297 (22.7) | 399 (24.9) | 65 (22.6) | 50 (19.9) | 136 (29.9) | 252 (24.5) |
| Other | 440 (25.3) | 863 (20.8) | 789 (20.2) | 307 (23.5) | 382 (23.9) | 48 (16.7) | 61 (24.3) | 137 (30.1) | 232 (22.6) |
| ART |  |  |  |  |  |  |  |  |  |
| No | 1341 (77.0) | 2991 (72.0) | 3041 (77.8) | 1024 (78.4) | 1061 (66.3) | 194 (67.6) | 177 (70.5) | 304 (66.8) | 765 (74.4) |
| Yes | 257 (14.8) | 820 (19.7) | 530 (13.6) | 136 (10.4) | 363 (22.7) | 66 (23.0) | 52 (20.7) | 79 (17.4) | 155 (15.1) |
| Unknow | 143 (8.2) | 341 (8.2) | 340 (8.7) | 146 (11.2) | 177 (11.1) | 27 (9.4) | 22 (8.8) | 72 (15.8) | 108 (10.5) |

T. m, *T. marneffei*; Mtb, mycobacterium tuberculosis; *T. pallidum,* Treponema pallidum.
