# Supplementary material for: Characteristics and risk factors for readmission in HIV-infected patients with Talaromyces marneffei infection
Source: PLoS Negl Trop Dis. 2023 Oct 10;17(10):e0011622. doi: 10.1371/journal.pntd.0011622 (PMC10564132; doi:10.1371/journal.pntd.0011622)
Supplement: S2 Table — (DOCX) [file pntd.0011622.s002.docx]

**S2 Table. Disease spectrum among HIV/AIDS patients with *T. marneffei* infection for three consecutive hospital admissions**

|  | | First admission | |  | Second admission | |  | Third admission | |  |
| --- | --- | --- | --- | --- | --- | --- | --- | --- | --- | --- |
| Complications | | Readmission | |  | Readmission | |  | Readmission | |  |
|  |  | No (n=1453) | Yes (n=288) | *p* | No (n=234) | Yes (n=54) | *p* | No (n=35) | Yes (n=19) | *p* |
| Mtb infection | No | 896 (61.7） | 134 (46.5) | <0.001 | 81 (34.6) | 17 (31.5) | 0.676 | 8 (22.9) | 4 (21.1) | 1.000 |
|  | Yes | 557 (38.3） | 154 (53.5) |  | 153 (65.4) | 37 (68.5) |  | 27 (77.1) | 15 (78.9) |  |
| Candida infection | No | 663 (45.6） | 125 (43.4) | 0.488 | 182 (77.8) | 41 (75.9) | 0.757 | 28 (80.0) | 16 (84.2) | 1.000 |
|  | Yes | 790 (54.4） | 163 (56.6) |  | 52 (22.2) | 13 (24.1) |  | 7 (20.0) | 3 (15.8) |  |
| IRIS | No | 1442 (99.2） | 281 (97.6) | 0.019 | 215 (91.9) | 46 (85.2) | 0.125 | 31 (88.6) | 18 (94.7) | 0.646 |
|  | Yes | 11 (0.8） | 7 (2.4) |  | 19 (8.1) | 8 (14.8) |  | 4 (11.4) | 1 (5.3) |  |
| Pneumonia | No | 341 (23.5） | 85 (29.5) | 0.029 | 141 (60.3) | 28 (51.9) | 0.248 | 21 (60.0) | 12 (63.2) | 1.000 |
|  | Yes | 1112 (76.5） | 203 (70.5) |  | 93 (39.7) | 26 (48.1) |  | 14 (40.0) | 7 (36.8) |  |
| Pneumocystis infection | No | 1234 (84.9） | 258 (89.6) | 0.039 | 220 (94.0) | 50 (92.6) | 0.754 | 34 (97.1) | 18 (94.7) | 1.000 |
|  | Yes | 219 (15.1） | 30 (10.4) |  | 14 (6.0) | 4 (7.4) |  | 1 (2.9) | 1 (5.3) |  |
| Bronchitis | No | 1446 (99.5） | 284 (98.6) | 0.093 | 233 (99.6) | 53 (98.1) | 0.339 | 33 (94.3) | 19 (100.0) | 0.535 |
|  | Yes | 7 (0.5) | 4 (1.4) |  | 1 (0.4) | 1 (1.9) |  | 2 (5.7) | 0 (0.0) |  |
| Hepatitis (B or C) | No | 1234 (84.9） | 236 (81.9) | 0.202 | 199 (85.0) | 43 (79.6) | 0.364 | 29 (82.9) | 14 (73.7) | 0.489 |
|  | Yes | 219 (15.1） | 52 (18.1) |  | 35 (15.0) | 11 (20.4) |  | 6 (17.1) | 5 (26.3) |  |
| Enteritis | No | 1374 (94.6） | 265 (92.0) | 0.092 | 221 (94.4) | 51 (94.4) | 1.000 | 34 (97.1) | 18 (94.7) | 1.000 |
|  | Yes | 79 (5.4） | 23 (8.0) |  | 13 (5.6) | 3 (5.6) |  | 1 (2.9) | 1 (5.3) |  |
| Herpesvirus infection | No | 1438 (99.0） | 283 (98.3) | 0.357 | 227 (97.0) | 53 (98.1) | 1.000 | 33 (94.3) | 19 (100.0) | 0.535 |
|  | Yes | 15 (1.0） | 5 (1.7) |  | 7 (3.0) | 1 (1.9) |  | 2 (5.7) | 0 (0.0) |  |
| Cryptococcus infection | No | 1444 (99.4） | 285 (99.0) | 0.431 | 232 (99.1) | 54 (100.0) | 1.000 | 35 (100.0) | 19 (100.0) | - |
|  | Yes | 9 (0.6) | 3 (1.0) |  | 2 (0.9) | 0 (0.0) |  | 0 (0.0) | 0 (0.0) |  |
| Hypoproteinemia | No | 1168 (80.4） | 240 (83.3) | 0.245 | 204 (87.2) | 47 (87.0) | 0.969 | 33 (94.3) | 17 (89.5) | 0.607 |
|  | Yes | 285 (19.6） | 48 (16.7) |  | 30 (12.8) | 7 (13.0) |  | 2 (5.7) | 2 (10.5) |  |
| Dermatitis | No | 1426 (98.1） | 285 (99.0) | 0.459 | 232 (99.1) | 53 (98.1) | 0.464 | 35 (100.0) | 19 (100.0) | - |
|  | Yes | 27 (1.9） | 3 (1.0) |  | 2 (0.9) | 1 (1.9) |  | 0 (0.0) | 0 (0.0) |  |
| Septic shock | No | 1363 (93.8） | 287 (99.7) | <0.001 | 227 (97.0) | 54 (100.0) | 0.355 | 35 (100.0) | 19 (100.0) | - |
|  | Yes | 90 (6.2） | 1 (0.3) |  | 7 (3.0) | 0 (0.0) |  | 0 (0.0) | 0 (0.0) |  |
| Hypertension | No | 1437 (98.9） | 285 (99.0) | 1.000 | 226 (96.6) | 52 (96.3) | 1.000 | 34 (97.1) | 19 (100.0) | 1.000 |
|  | Yes | 16 (1.1） | 3 (1.0) |  | 8 (3.4) | 2 (3.7) |  | 1 (2.9) | 0 (0.0) |  |
| Diabetes | No | 1426 (98.1） | 285 (99.0) | 0.459 | 232 (99.1) | 53 (98.1) | 0.464 | 35 (100.0) | 19 (100.0) | - |
|  | Yes | 27 (1.9） | 3 (1.0) |  | 2 (0.9) | 1 (1.9) |  | 0 (0.0) | 0 (0.0) |  |
| Electrolyte disturbances | No | 1160 (79.8） | 228 (79.2) | 0.797 | 191 (81.6) | 46 (85.2) | 0.545 | 29 (82.9) | 15 (78.9) | 0.728 |
|  | Yes | 293 (20.2） | 60 (20.8) |  | 43 (18.4) | 8 (14.8) |  | 6 (17.1) | 4 (21.1) |  |
| Respiratory failure | No | 1395 (96.0） | 282 (97.9) | 0.116 | 225 (96.2) | 53 (98.1) | 0.694 | 34 (97.1) | 19 (100.0) | 1.000 |
|  | Yes | 58 (4.0） | 6 (2.1) |  | 9 (3.8) | 1 (1.9) |  | 1 (2.9) | 0 (0.0) |  |
| *T. pallidum* infection | No | 1400 (96.4） | 280 (97.2) | 0.599 | 232 (99.1) | 53 (98.1) | 0.464 | 35 (100.0) | 19 (100.0) | - |
|  | Yes | 53 (3.6） | 8 (2.8) |  | 2 (0.9) | 1 (1.9) |  | 0 (0.0) | 0 (0.0) |  |
| Cytomegalovirus infection | No | 1192 (82.0） | 237 (82.3) | 0.918 | 204 (87.2) | 45 (83.3) | 0.505 | 27 (77.1) | 15 (78.9) | 1.000 |
|  | Yes | 261 (18.0） | 51 (17.7) |  | 30 (12.8) | 9 (16.7) |  | 8 (22.9) | 4 (21.1) |  |
| Tumour | No | 1448 (99.7） | 287 (99.7) | 1.000 | 233 (99.6) | 53 (98.1) | 0.339 | 34 (97.1) | 19 (100.0) | 1.000 |
|  | Yes | 5 (0.3) | 1 (0.3) |  | 1 (0.4) | 1 (1.9) |  | 1 (2.9) | 0 (0.0) |  |

Mtb, mycobacterium tuberculosis; *T. pallidum*, Treponema pallidum.
