# Supplementary material for: Characteristics and risk factors for readmission in HIV-infected patients with Talaromyces marneffei infection
Source: PLoS Negl Trop Dis. 2023 Oct 10;17(10):e0011622. doi: 10.1371/journal.pntd.0011622 (PMC10564132; doi:10.1371/journal.pntd.0011622)
Supplement: S3 Table — (DOCX) [file pntd.0011622.s003.docx]

**S3 Table. Laboratory test results among HIV/AIDS patients with *T. marneffei* infection for three consecutive hospital admissions**

|  | First admission | | |  | Second admission | | |  | Third admission | | |
| --- | --- | --- | --- | --- | --- | --- | --- | --- | --- | --- | --- |
| Complications | Readmission | | |  | Readmission | | |  | Readmission | | |
|  | No (n=1453) | Yes (n=288) | *p* |  | No (n=234) | Yes (n=54) | *p* |  | No (n=35) | Yes (n=19) | *p* |
| CD3+T cell (cells/ul) |  |  | 0.095 |  |  |  | 1.000 |  |  |  | 0.734 |
| ≥690 | 186 (13.7) | 27 (9.9) |  |  | 57 (33.9) | 12 (33.3) |  |  | 8 (30.8) | 6 (40.0) |  |
| <690 | 1170 (86.3) | 245 (90.1) |  |  | 111 (66.1) | 24 (66.7) |  |  | 18 (69.2) | 9 (60.0) |  |
| CD4/CD8 ratio |  |  | 1.000* |  |  |  | 0.323* |  |  |  | 1.000 |
| ≥1 | 14 (1.0) | 3 (1.1) |  |  | 1 (0.6) | 1 (2.8) |  |  | 1 (3.8) | 0 (0.0) |  |
| ＜1 | 1342 (99.0) | 269 (98.9) |  |  | 167 (99.4) | 35 (97.2) |  |  | 25 (96.2) | 15 (100.0) |  |
| CD4+T cell (cells/ul) |  |  | 0.344 |  |  |  | 0.269 |  |  |  | 0.695 |
| 200-349 | 28 (2.1) | 2 (0.7) |  |  | 8 (4.8) | 3 (8.3) |  |  | 2 (7.7) | 0 (0.0) |  |
| <200 | 1306 (96.3) | 267 (98.2) |  |  | 156 (92.9) | 31 (86.1) |  |  | 23 (88.5) | 15 (100.0) |  |
| >350 | 22 (1.6) | 3 (1.1) |  |  | 4 (2.4) | 2 (5.6) |  |  | 1 (3.8) | 0 (0.0) |  |
| CD8+T cell (cells/ul) |  |  | 0.309 |  |  |  | 0.653 |  |  |  | 0.693 |
| ≥190 | 820 (60.5) | 155 (57.0) |  |  | 134 (79.8) | 27 (75.0) |  |  | 22 (84.6) | 12 (80.0) |  |
| <190 | 536 (39.5) | 117 (43.0) |  |  | 34 (20.2) | 9 (25.0) |  |  | 4 (15.4) | 3 (20.0) |  |
| ALT (U/L) |  |  | 0.348 |  |  |  | 0.490 |  |  |  | 0.436 |
| ≤40 | 564 (61.0) | 97 (57.1) |  |  | 117 (81.3) | 28 (75.7) |  |  | 19 (82.6) | 10 (66.7) |  |
| >40 | 360 (39.0) | 73 (42.9) |  |  | 27 (18.8) | 9 (24.3) |  |  | 4 (17.4) | 5 (33.3) |  |
| Ca (mmol/L) |  |  | 0.404 |  |  |  | 0.374 |  |  |  | 0.852 |
| 2.11-2.52 | 267 (20.0) | 44 (16.4) |  |  | 74 (35.4) | 20 (41.7) |  |  | 16 (51.6) | 8 (44.4) |  |
| ＜2.11 | 1041 (78.1) | 219 (81.7) |  |  | 133 (63.6) | 27 (56.3) |  |  | 14 (45.2) | 10 (55.6) |  |
| ＞2.52 | 25 (1.9) | 5 (1.9) |  |  | 2 (1.0) | 1 (2.1) |  |  | 1 (3.2) | 0 (0.0) |  |
| K (mmol/L) |  |  | 0.834 |  |  |  | 0.154 |  |  |  | 0.721 |
| 3.5-5.5 | 818 (61.0) | 163 (59.7) |  |  | 117 (53.9) | 25 (48.1) |  |  | 16 (50.0) | 11 (61.1) |  |
| ＜3.5 | 493 (36.8) | 105 (38.5) |  |  | 100 (46.1) | 26 (50.0) |  |  | 15 (46.9) | 7 (38.9) |  |
| ＞5.5 | 29 (2.2) | 5 (1.8) |  |  | 0 (0.0) | 1 (1.9) |  |  | 1 (3.1) | 0 (0.0) |  |
| TG (mmol/L) |  |  | 0.825 |  |  |  | 0.336 |  |  |  | 0.475 |
| 0-1.7 | 609 (54.8) | 125 (55.8) |  |  | 102 (62.6) | 18 (52.9) |  |  | 9 (39.1) | 6 (54.5) |  |
| ＞1.7 | 502 (45.2) | 99 (44.2) |  |  | 61 (37.4) | 16 (47.1) |  |  | 14 (60.9) | 5 (45.5) |  |
| AST (U/L) |  |  | 0.088 |  |  |  | 1.000 |  |  |  | 1.000 |
| ≤40 | 234 (25.3) | 54 (31.8) |  |  | 93 (64.6) | 24 (64.9) |  |  | 16 (69.6) | 10 (66.7) |  |
| >40 | 690 (74.7) | 116 (68.2) |  |  | 51 (35.4) | 13 (35.1) |  |  | 7 (30.4) | 5 (33.3) |  |
| UA (umol/L) |  |  | 0.053 |  |  |  | 0.354 |  |  |  | 0.471 |
| 155-357 | 821 (61.0) | 175 (64.1) |  |  | 122 (56.7) | 35 (67.3) |  |  | 18 (56.3) | 10 (55.6) |  |
| ＜155 | 282 (21.0) | 65 (23.8) |  |  | 21 (9.8) | 5 (9.6) |  |  | 3 (9.4) | 0 (0.0) |  |
| ＞357 | 242 (18.0) | 33 (12.1) |  |  | 72 (33.5) | 12 (23.1) |  |  | 11 (34.4) | 8 (44.4) |  |
| CK (U/L) |  |  | 0.174 |  |  |  | 1.000 |  |  |  | 0.528 |
| ≤200 | 1068 (84.6) | 221 (88.0) |  |  | 170 (95.5) | 38 (97.4) |  |  | 23 (92.0) | 14 (100.0) |  |
| ＞200 | 195 (15.4) | 30 (12.0) |  |  | 8 (4.5) | 1 (2.6) |  |  | 2 (8.0) | 0 (0.0) |  |
| Hb (g/L) |  |  | 0.355* |  |  |  | 0.679 |  |  |  | 0.704 |
| 115-150 | 200 (14.6) | 34 (12.1) |  |  | 36 (16.0) | 7 (13.2) |  |  | 5 (14.3) | 4 (21.1) |  |
| ＜115 | 1159 (84.4) | 245 (87.5) |  |  | 189 (84.0) | 46 (86.8) |  |  | 30 (85.7) | 15 (78.9) |  |
| ＞150 | 15 (1.1) | 1 (0.4) |  |  | 0 (0.0) | 0 (0.0) |  |  | 0 (0.0) | 0 (0.0) |  |
| PLT (10^9^/L) |  |  | 0.792 |  |  |  | 0.544 |  |  |  | 1.000 |
| 125-350 | 561 (40.8) | 120 (42.9) |  |  | 134 (59.6) | 33 (62.3) |  |  | 20 (57.1) | 12 (63.2) |  |
| ＜125 | 757 (55.1) | 148 (52.9) |  |  | 73 (32.4) | 14 (26.4) |  |  | 12 (34.3) | 6 (31.6) |  |
| ＞350 | 56 (4.1) | 12 (4.3) |  |  | 18 (8.0) | 6 (11.3) |  |  | 3 (8.6) | 1 (5.3) |  |
| LYMPH (10^9^/L) |  |  | 0.096 |  |  |  | 0.563 |  |  |  | 0.550 |
| 1.1-3.2 | 159 (11.6) | 26 (9.3) |  |  | 52 (23.1) | 15 (28.3) |  |  | 13 (37.1) | 5 (26.3) |  |
| ＜1.1 | 1174 (85.4) | 251 (89.6) |  |  | 169 (75.1) | 38 (71.7) |  |  | 22 (62.9) | 14 (73.7) |  |
| ＞3.2 | 41 (3.0) | 3 (1.1) |  |  | 4 (1.8) | 0 (0.0) |  |  | 0 (0.0) | 0 (0.0) |  |
| LDL (mmol/L) |  |  | 0.042 |  |  |  | 1.000 |  |  |  | 1.000 |
| ≤3.37 | 1063 (95.7) | 206 (92.4) |  |  | 149 (92.0) | 31 (91.2) |  |  | 21 (91.3) | 10 (90.9) |  |
| ＞3.37 | 48 (4.3) | 17 (7.6) |  |  | 13 (8.0) | 3 (8.8) |  |  | 2 (8.7) | 1 (9.1) |  |
| HDL (mmol/L) |  |  | 0.142 |  |  |  | 0.820 |  |  |  | 0.189 |
| 1.1-1.74 | 21 (1.9) | 9 (4.0) |  |  | 18 (11.0) | 5 (14.7) |  |  | 0 (0.0) | 2 (18.2) |  |
| ＜1.1 | 1091 (98.0) | 216 (96.0) |  |  | 140 (85.9) | 28 (82.4) |  |  | 21 (91.3) | 9 (81.8) |  |
| ＞1.74 | 1 (0.1) | 0 (0.0) |  |  | 5 (3.1) | 1 (2.9) |  |  | 2 (8.7) | 0 (0.0) |  |
| CREA (umol/L) |  |  | 0.088 |  |  |  | 0.114 |  |  |  | 0.322 |
| 41-81 | 787 (58.5) | 166 (60.8) |  |  | 119 (55.3) | 37 (71.2) |  |  | 14 (43.8) | 12 (66.7) |  |
| ＜41 | 63 (4.7) | 20 (7.3) |  |  | 12 (5.6) | 1 (1.9) |  |  | 4 (12.5) | 1 (5.6) |  |
| ＞81 | 495 (36.8) | 87 (31.9) |  |  | 84 (39.1) | 14 (26.9) |  |  | 14 (43.8) | 5 (27.8) |  |
| UREA (mmol/L) |  |  | 0.005 |  |  |  | 0.280 |  |  |  | 1.000 |
| 2.6-7.5 | 946 (70.2) | 216 (79.1) |  |  | 159 (73.3) | 42 (80.8) |  |  | 22 (68.8) | 12 (66.7) |  |
| ＜2.6 | 97 (7.2) | 19 (7.0) |  |  | 22 (10.1) | 6 (11.5) |  |  | 4 (12.5) | 2 (11.1) |  |
| ＞7.5 | 304 (22.6) | 38 (13.9) |  |  | 36 (16.6) | 4 (7.7) |  |  | 6 (18.8) | 4 (22.2) |  |
| LDH (U/L) |  |  | 0.183 |  |  |  | 0.609 |  |  |  | 0.833 |
| 120-250 | 238 (18.7) | 60 (23.3) |  |  | 84 (44.7) | 22 (51.2) |  |  | 15 (55.6) | 9 (64.3) |  |
| ＜120 | 5 (0.4) | 1 (0.4) |  |  | 6 (3.2) | 0 (0.0) |  |  | 1 (3.7) | 0 (0.0) |  |
| ＞250 | 1027 (80.9) | 196 (76.3) |  |  | 98 (52.1) | 21 (48.8) |  |  | 11 (40.7) | 5 (35.7) |  |
| TBIL (umol/L) |  |  | 0.764 |  |  |  | 0.677 |  |  |  | 0.681 |
| ≤21 | 1007 (73.4) | 203 (74.4) |  |  | 179 (82.9) | 44 (86.3) |  |  | 28 (87.5) | 14 (82.4) |  |
| ＞21 | 365 (26.6) | 70 (25.6) |  |  | 37 (17.1) | 7 (13.7) |  |  | 4 (12.5) | 3 (17.6) |  |
| AST/ALT ratio |  |  | 0.147 |  |  |  | 0.018 |  |  |  | 0.590 |
| 0.8-1.5 | 303 (22.1) | 75 (27.5) |  |  | 95 (44.0) | 20 (39.2) |  |  | 10 (31.3) | 6 (35.3) |  |
| ＜0.8 | 47 (3.4) | 8 (2.9) |  |  | 11 (5.1) | 9 (17.6) |  |  | 3 (9.4) | 3 (17.6) |  |
| ＞1.5 | 1022 (74.5) | 190 (69.6) |  |  | 110 (50.9) | 22 (43.1) |  |  | 19 (59.4) | 8 (47.1) |  |
| Ccr (mol/min) |  |  | 0.084 |  |  |  | 1.000 |  |  |  | 1.000 |
| 72-172 | 242 (32.5) | 64 (41.3) |  |  | 37 (34.3) | 6 (33.3) |  |  | 3 (21.4) | 2 (28.6) |  |
| ＜72 | 499 (67.0) | 91 (58.7) |  |  | 71 (65.7) | 12 (66.7) |  |  | 11 (78.6) | 5 (71.4) |  |
| ＞172 | 4 (0.5) | 0 (0.0) |  |  | 0 (0.0) | 0 (0.0) |  |  | 0 (0.0) | 0 (0.0) |  |
| WBC (10^9^/L) |  |  | 0.022 |  |  |  | 0.937 |  |  |  | 0.538 |
| 3.5-9.5 | 752 (54.7) | 137 (48.9) |  |  | 117 (52.0) | 29 (54.7) |  |  | 22 (62.9) | 9 (47.4) |  |
| ＜3.5 | 498 (36.2) | 125 (44.6) |  |  | 95 (42.2) | 21 (39.6) |  |  | 11 (31.4) | 9 (47.4) |  |
| ＞9.5 | 124 (9.0) | 18 (6.4) |  |  | 13 (5.8) | 3 (5.7) |  |  | 2 (5.7) | 1 (5.3) |  |
| NEUT (10^9^/L) |  |  | 0.04 |  |  |  | 0.918 |  |  |  | 0.134 |
| 40-75 | 913 (66.4) | 188 (67.1) |  |  | 127 (56.4) | 32 (60.4) |  |  | 26 (74.3) | 9 (47.4) |  |
| ＜40 | 265 (19.3) | 66 (23.6) |  |  | 79 (35.1) | 17 (32.1) |  |  | 7 (20.0) | 8 (42.1) |  |
| ＞75 | 196 (14.3) | 26 (9.3) |  |  | 19 (8.4) | 4 (7.5) |  |  | 2 (5.7) | 2 (10.5) |  |
