# Supplementary material for: Characteristics and risk factors for readmission in HIV-infected patients with Talaromyces marneffei infection
Source: PLoS Negl Trop Dis. 2023 Oct 10;17(10):e0011622. doi: 10.1371/journal.pntd.0011622 (PMC10564132; doi:10.1371/journal.pntd.0011622)
Supplement: S4 Table — (DOCX) [file pntd.0011622.s004.docx]

**S4 Table. Disease spectrum among HIV/AIDS patients with non-*T. marneffei* infection during three consecutive hospital admissions**

|  | | First admission | |  | Second admission | |  | Third admission | |  |
| --- | --- | --- | --- | --- | --- | --- | --- | --- | --- | --- |
| Complications | | Readmission | |  | Readmission | |  | Readmission | |  |
|  |  | No (n=7943) | Yes (n=3262) | *p* | No (n=2012) | Yes (n=1250) | *p* | No (n=645) | Yes (n=605) | *p* |
| Mtb infection | No | 5641 (71.0) | 2123 (65.1) | <0.001 | 1235 (61.4) | 757 (60.6) | 0.64 | 385 (59.7) | 383 (63.3） | 0.189 |
|  | Yes | 2302 (29.0) | 1139 (34.9) |  | 777 (38.6) | 493 (39.4) |  | 260 (40.3) | 222 (36.7） |  |
| Candida infection | No | 5846 (73.6) | 2401 (73.6) | 0.988 | 1656 (82.3) | 1070 (85.6) | 0.014 | 563 (87.3) | 535 (88.4） | 0.537 |
|  | Yes | 2097 (26.4) | 861 (26.4) |  | 356 (17.7) | 180 (14.4) |  | 82 (12.7) | 70 (11.6） |  |
| IRIS | No | 7905 (99.5) | 3238 (99.3) | 0.096 | 1934 (96.1) | 1205 (96.4) | 0.687 | 624 (96.7) | 588 (97.2） | 0.646 |
|  | Yes | 38 (0.5) | 24 (0.7) |  | 78 (3.9) | 45 (3.6) |  | 21 (3.3) | 17 (2.8） |  |
| Pneumonia | No | 4308 (54.2) | 1852 (56.8) | 0.014 | 1285 (63.9） | 903 (72.2) | <0.001 | 439 (68.1) | 459 (75.9) | 0.002 |
|  | Yes | 3635 (45.8) | 1410 (43.2) |  | 727 (36.1) | 347 (27.8) |  | 206 (31.9) | 146 (24.1） |  |
| Pneumocystis infection | No | 7160 (90.1) | 2988 (91.6) | 0.016 | 1894 (94.1) | 1217 (97.4) | <0.001 | 619 (96.0) | 593 (98.0） | 0.035 |
|  | Yes | 783 (9.9) | 274 (8.4) |  | 118 (5.9) | 33 (2.6) |  | 26 (4.0) | 12 (2.0） |  |
| Bronchitis | No | 7836 (98.7) | 3209 (98.4) | 0.261 | 1986 (98.7) | 1233 (98.6) | 0.869 | 634 (98.3) | 596 (98.5） | 0.759 |
|  | Yes | 107 (1.3) | 53 (1.6) |  | 26 (1.3) | 17 (1.4) |  | 11 (1.7) | 9 (1.5） |  |
| Hepatitis (B or C) | No | 7057 (88.8) | 2818 (86.4) | <0.001 | 1791 (89.0) | 1098 (87.8) | 0.305 | 583 (90.4) | 536 (88.6） | 0.301 |
|  | Yes | 886 (11.2) | 444 (13.6) |  | 221 (11.0) | 152 (12.2) |  | 62 (9.6) | 69 (11.4） |  |
| Enteritis | No | 7667 (96.5) | 3125 (95.8) | 0.065 | 1937 (96.3) | 1212 (97.0) | 0.296 | 630 (97.7) | 589 (97.4） | 0.717 |
|  | Yes | 276 (3.5) | 137 (4.2) |  | 75 (3.7) | 38 (3.0) |  | 15 (2.3) | 16 (2.6） |  |
| Herpesvirus infection | No | 7748 (97.5) | 3190 (97.8) | 0.433 | 1961 (97.5) | 1217 (97.4) | 0.854 | 630 (97.7) | 594 (98.2） | 0.53 |
|  | Yes | 195 (2.5) | 72 (2.2) |  | 51 (2.5) | 33 (2.6) |  | 15 (2.3) | 11 (1.8） |  |
| Cryptococcus infection | No | 7787 (98.0) | 3179 (97.5) | 0.054 | 1970 (97.9) | 1211 (96.9) | 0.065 | 620 (96.1) | 585 (96.7） | 0.589 |
|  | Yes | 156 (2.0) | 83 (2.5) |  | 42 (2.1) | 39 (3.1) |  | 25 (3.9) | 20 (3.3） |  |
| Hypoproteinemia | No | 7360 (92.7) | 3088 (94.7) | <0.001 | 1885 (93.7) | 1205 (96.4) | 0.001 | 604 (93.6) | 577 (95.4） | 0.181 |
|  | Yes | 583 (7.3) | 174 (5.3) |  | 127 (6.3) | 45 (3.6) |  | 41 (6.4) | 28 (4.6） |  |
| Dermatitis | No | 7692 (96.8) | 3129 (95.9) | 0.015 | 1923 (95.6) | 1192 (95.4) | 0.772 | 616 (95.5) | 571 (94.4） | 0.364 |
|  | Yes | 251 (3.2) | 133 (4.1) |  | 89 (4.4) | 58 (4.6) |  | 29 (4.5) | 34 (5.6） |  |
| Septic shock | No | 7813 (98.4) | 3260 (99.9) | <0.001 | 1982 (98.5) | 1250 (100.0) | <0.001 | 631 (97.8) | 602 (99.5） | 0.011 |
|  | Yes | 130 (1.6) | 2 (0.1) |  | 30 (1.5) | 0 (0.0) |  | 14 (2.2) | 3 (0.5） |  |
| Hypertension | No | 7419 (93.4) | 2980 (91.4) | <0.001 | 1834 (91.2) | 1098 (87.8) | 0.002 | 562 (87.1) | 528 (87.3） | 0.941 |
|  | Yes | 524 (6.6) | 282 (8.6) |  | 178 (8.8) | 152 (12.2) |  | 83 (12.9) | 77 (12.7） |  |
| Diabetes | No | 7692 (96.8) | 3129 (95.9) | 0.015 | 1923 (95.6) | 1192 (95.4) | 0.772 | 616 (95.5) | 571 (94.4） | 0.364 |
|  | Yes | 251 (3.2) | 133 (4.1) |  | 89 (4.4) | 58 (4.6) |  | 29 (4.5) | 34 (5.6） |  |
| Electrolyte disturbances | No | 7114 (89.6) | 3025 (92.7) | <0.001 | 1822 (90.6) | 1167 (93.4) | 0.005 | 573 (88.8) | 574 (94.9） | <0.001 |
|  | Yes | 829 (10.4) | 237 (7.3) |  | 190 (9.4) | 83 (6.6) |  | 72 (11.2) | 31 (5.1） |  |
| Respiratory failure | No | 7633 (96.1) | 3230 (99.0) | <0.001 | 1954 (97.1) | 1241 (99.3) | <0.001 | 625 (96.9) | 601 (99.3） | 0.002 |
|  | Yes | 310 (3.9) | 32 (1.0) |  | 58 (2.9) | 9 (0.7) |  | 20 (3.1) | 4 (0.7） |  |
| *T. pallidum* infection | No | 7650 (96.3) | 3161 (96.9) | 0.121 | 1979 (98.4) | 1234 (98.7) | 0.411 | 640 (99.2) | 593 (98.0） | 0.065 |
|  | Yes | 293 (3.7) | 101 (3.1) |  | 33 (1.6) | 16 (1.3) |  | 5 (0.8) | 12 (2.0） |  |
| Cytomegalovirus infection | No | 7394 (93.1) | 3095 (94.9) | <0.001 | 1899 (94.4) | 1196 (95.7) | 0.102 | 626 (97.1) | 584 (96.5） | 0.598 |
|  | Yes | 549 (6.9) | 167 (5.1) |  | 113 (5.6) | 54 (4.3) |  | 19 (2.9) | 21 (3.5） |  |
| Tumour | No | 7880 (99.2) | 3244 (99.4) | 0.170 | 2001 (99.5) | 1247 (99.8) | 0.193 | 641 (99.4) | 601 (99.3） | 1.000* |
|  | Yes | 63 (0.8) | 18 (0.6) |  | 11 (0.5) | 3 (0.2) |  | 4 (0.6) | 4 (0.7） |  |

Mtb, mycobacterium tuberculosis; *T. pallidum*, Treponema pallidum.
