# Supplementary material for: Characteristics and risk factors for readmission in HIV-infected patients with Talaromyces marneffei infection
Source: PLoS Negl Trop Dis. 2023 Oct 10;17(10):e0011622. doi: 10.1371/journal.pntd.0011622 (PMC10564132; doi:10.1371/journal.pntd.0011622)
Supplement: S5 Table — (DOCX) [file pntd.0011622.s005.docx]

**S5 Table. Laboratory test results among HIV/AIDS patients with non-*T. marneffei* infection during three consecutive hospital admissions**

|  | First admission | | |  | Second admission | | |  | Third admission | | |
| --- | --- | --- | --- | --- | --- | --- | --- | --- | --- | --- | --- |
| Complications | Readmission | | |  | Readmission | | |  | Readmission | | |
|  | No (n=1453) | Yes (n=288) | *p* |  | No (n=234) | Yes (n=54) | *p* |  | No (n=35) | Yes (n=19) | *p* |
| CD3+T cell (cells/ul) |  |  | 0.038 |  |  |  | 0.623 |  |  |  | 0.744 |
| ≥690 | 3925 (70.2) | 1663 (29.8) |  |  | 920 (62.0) | 565 (38.0) |  |  | 330 (52.2) | 302 (47.8) |  |
| <690 | 3239 (72.1) | 1251 (27.9) |  |  | 700 (62.9) | 412 (37.1) |  |  | 201 (53.5) | 175 (46.5) |  |
| CD4/CD8 ratio |  |  | 0.005 |  |  |  | 0.002 |  |  |  | 0.035 |
| ≥1 | 773 (74.8) | 260 (25.2) |  |  | 241 (69.9) | 104 (30.1) |  |  | 85 (61.2) | 54 (38.8) |  |
| ＜1 | 6391 (70.7) | 2654 (29.3) |  |  | 1379 (61.2) | 873 (38.8) |  |  | 446 (51.3) | 423 (48.7) |  |
| CD4+T cell (cells/ul) |  |  | 0.93 |  |  |  | <0.001 |  |  |  | 0.132 |
| 200-349 | 1308 (70.7) | 541 (29.3) |  |  | 289 (53.8) | 248 (46.2) |  |  | 120 (48.2) | 129 (51.8) |  |
| <200 | 4071 (71.1) | 1653 (28.9) |  |  | 872 (63.9) | 493 (36.1) |  |  | 251 (52.5) | 227 (47.5) |  |
| >350 | 1785 (71.3) | 720 (28.7) |  |  | 459 (66.0) | 236 (34.0) |  |  | 160 (56.9) | 121 (43.1) |  |
| CD8+T cell (cells/ul) |  |  | 0.004 |  |  |  | 0.001 |  |  |  | 0.132 |
| ≥190 | 6374 (70.6) | 2650 (29.4) |  |  | 1441 (61.3) | 909 (38.7) |  |  | 473 (51.9) | 439 (48.1) |  |
| <190 | 790 (75.0) | 264 (25.0) |  |  | 179 (72.5) | 68 (27.5) |  |  | 58 (60.4) | 38 (39.6) |  |
| ALT (U/L) |  |  | 0.248 |  |  |  | 0.174 |  |  |  | 0.573 |
| ≤40 | 4675 (73.1) | 1722 (26.9) |  |  | 1275 (62.7) | 758 (37.3) |  |  | 445 (52.8) | 398 (47.2) |  |
| >40 | 1151 (74.5) | 393 (25.5) |  |  | 266 (66.3) | 135 (33.7) |  |  | 73 (55.7) | 58 (44.3) |  |
| Ca (mmol/L) |  |  | <0.001 |  |  |  | 0.092 |  |  |  | 0.070 |
| 2.11-2.52 | 3646 (69.3) | 1613 (30.7) |  |  | 1019 (60.1) | 677 (39.9) |  |  | 336 (48.8) | 353 (51.2) |  |
| ＜2.11 | 3427 (72.7) | 1287 (27.3) |  |  | 757 (64.1) | 424 (35.9) |  |  | 225 (55.7) | 179 (44.3) |  |
| ＞2.52 | 193 (77.8) | 55 (22.2) |  |  | 44 (62.9) | 26 (37.1) |  |  | 20 (46.5) | 23 (53.5) |  |
| K (mmol/L) |  |  | 0.21 |  |  |  | 0.016 |  |  |  | 0.130 |
| 3.5-5.5 | 5253 (70.5) | 2197 (29.5) |  |  | 1302 (60.1) | 866 (39.9) |  |  | 443 (52.3) | 404 (47.7) |  |
| ＜3.5 | 1956 (72.0) | 760 (28.0) |  |  | 496 (65.6) | 260 (34.4) |  |  | 134 (49.3) | 138 (50.7) |  |
| ＞5.5 | 143 (74.1) | 50 (25.9) |  |  | 43 (67.2) | 21 (32.8) |  |  | 10 (34.5) | 19 (65.5) |  |
| TG (mmol/L) |  |  | 0.619 |  |  |  | 0.701 |  |  |  | 0.408 |
| 0-1.7 | 3581 (70.9) | 1471 (29.1) |  |  | 778 (61.1) | 496 (38.9) |  |  | 231 (48.2) | 248 (51.8) |  |
| ＞1.7 | 1666 (71.5) | 665 (28.5) |  |  | 444 (62.0) | 272 (38.0) |  |  | 145 (51.6) | 136 (48.4) |  |
| AST (U/L) |  |  | <0.001 |  |  |  | 0.058 |  |  |  | 0.088 |
| ≤40 | 4128 (71.7) | 1627 (28.3) |  |  | 1167 (62.3) | 707 (37.7) |  |  | 408 (51.8) | 379 (48.2) |  |
| >40 | 1698 (77.7) | 488 (22.3) |  |  | 374 (66.8) | 186 (33.2) |  |  | 110 (58.8) | 77 (41.2) |  |
| UA (umol/L) |  |  | 0.008 |  |  |  | 0.773 |  |  |  | 0.327 |
| 155-357 | 4591 (71.1) | 1869 (28.9) |  |  | 1086 (61.6) | 676 (38.4) |  |  | 341 (50.4) | 336 (49.6) |  |
| ＜155 | 761 (74.8) | 257 (25.2) |  |  | 113 (64.2) | 63 (35.8) |  |  | 29 (61.7) | 18 (38.3) |  |
| ＞357 | 2017 (69.6) | 879 (30.4) |  |  | 640 (61.4) | 403 (38.6) |  |  | 215 (50.9) | 207 (49.1) |  |
| CK (U/L) |  |  | 0.011 |  |  |  | 0.262 |  |  |  | 0.01 |
| ≤200 | 5668 (71.2) | 2295 (28.8) |  |  | 1373 (61.8) | 849 (38.2) |  |  | 428 (49.9) | 429 (50.1) |  |
| ＞200 | 847 (74.8) | 285 (25.2) |  |  | 179 (65.3) | 95 (34.7) |  |  | 73 (62.9) | 43 (37.1) |  |
| Hb (g/L) |  |  | 0.008 |  |  |  | 0.048* |  |  |  | 0.005 |
| 115-150 | 2797 (70.6) | 1164 (29.4) |  |  | 683 (60.6) | 444 (39.4) |  |  | 232 (48.8) | 243 (51.2) |  |
| ＜115 | 4338 (70.3) | 1834 (29.7) |  |  | 1134 (61.2) | 720 (38.8) |  |  | 344 (50.9) | 332 (49.1) |  |
| ＞150 | 393 (76.8) | 119 (23.2) |  |  | 93 (71.5) | 37 (28.5) |  |  | 34 (73.9) | 12 (26.1) |  |
| PLT (10^9^/L) |  |  | 0.027 |  |  |  | 0.301 |  |  |  | 0.633 |
| 125-350 | 5507 (70.1) | 2347 (29.9) |  |  | 1407 (60.6) | 913 (39.4) |  |  | 460 (50.8) | 446 (49.2) |  |
| ＜125 | 1163 (73.5) | 420 (26.5) |  |  | 294 (64.3) | 163 (35.7) |  |  | 90 (53.9) | 77 (46.1) |  |
| ＞350 | 858 (71.0) | 350 (29.0) |  |  | 209 (62.6) | 125 (37.4) |  |  | 60 (48.4) | 64 (51.6) |  |
| LYMPH (10^9^/L) |  |  | 0.025 |  |  |  | 0.033 |  |  |  | 0.109 |
| 1.1-3.2 | 3602 (69.6) | 1574 (30.4) |  |  | 971 (59.5) | 661 (40.5) |  |  | 342 (51.0) | 329 (49.0) |  |
| ＜1.1 | 3611 (71.6) | 1433 (28.4) |  |  | 863 (63.0) | 506 (37.0) |  |  | 243 (49.7) | 246 (50.3) |  |
| ＞3.2 | 315 (74.1) | 110 (25.9) |  |  | 76 (69.1) | 34 (30.9) |  |  | 25 (67.6) | 12 (32.4) |  |
| LDL (mmol/L) |  |  | 0.061 |  |  |  | 0.153 |  |  |  | 0.410 |
| ≤3.37 | 4551 (71.5) | 1814 (28.5) |  |  | 1052 (62.1) | 643 (37.9) |  |  | 326 (50.2) | 324 (49.8) |  |
| ＞3.37 | 690 (68.6) | 316 (31.4) |  |  | 169 (57.5) | 125 (42.5) |  |  | 50 (45.5) | 60 (54.5) |  |
| HDL (mmol/L) |  |  | 0.177 |  |  |  | 0.879 |  |  |  | 0.248 |
| 1.1-1.74 | 951 (69.6) | 415 (30.4) |  |  | 338 (61.2) | 214 (38.8) |  |  | 116 (47.3) | 129 (52.7) |  |
| ＜1.1 | 4093 (71.5) | 1628 (28.5) |  |  | 811 (61.6) | 505 (38.4) |  |  | 236 (51.6) | 221 (48.4) |  |
| ＞1.74 | 203 (67.9) | 96 (32.1) |  |  | 73 (59.3) | 50 (40.7) |  |  | 24 (41.4) | 34 (58.6) |  |
| CREA (umol/L) |  |  | 0.814 |  |  |  | 0.595 |  |  |  | 0.023 |
| 41-81 | 4625 (71.2) | 1867 (28.8) |  |  | 1094 (61.8) | 677 (38.2) |  |  | 318 (47.9) | 346 (52.1) |  |
| ＜41 | 308 (71.1) | 125 (28.9) |  |  | 86 (65.6) | 45 (34.4) |  |  | 26 (63.4) | 15 (36.6) |  |
| ＞81 | 2436 (70.6) | 1013 (29.4) |  |  | 659 (61.1) | 420 (38.9) |  |  | 241 (54.6) | 200 (45.4) |  |
| UREA (mmol/L) |  |  | 0.06 |  |  |  | 0.295 |  |  |  | 0.172 |
| 2.6-7.5 | 5705 (71.0) | 2332 (29.0) |  |  | 1361 (60.8) | 877 (39.2) |  |  | 425 (50.4) | 418 (49.6) |  |
| ＜2.6 | 604 (68.4) | 279 (31.6) |  |  | 167 (63.7) | 95 (36.3) |  |  | 41 (45.6) | 49 (54.4) |  |
| ＞7.5 | 1074 (73.0) | 398 (27.0) |  |  | 315 (64.2) | 176 (35.8) |  |  | 123 (56.2) | 96 (43.8) |  |
| LDH (U/L) |  |  | 0.056 |  |  |  | 0.045 |  |  |  | 0.273 |
| 120-250 | 3302 (70.8) | 1365 (29.2) |  |  | 863 (60.2) | 571 (39.8) |  |  | 299 (49.7) | 303 (50.3) |  |
| ＜120 | 59 (64.1) | 33 (35.9) |  |  | 34 (58.6) | 24 (41.4) |  |  | 16 (50.0) | 16 (50.0) |  |
| ＞250 | 3239 (72.5) | 1231 (27.5) |  |  | 684 (65.0) | 369 (35.0) |  |  | 195 (55.1) | 159 (44.9) |  |
| TBIL (umol/L) |  |  | 0.246 |  |  |  | 0.541 |  |  |  | 0.015 |
| ≤21 | 6686 (70.5) | 2794 (29.5) |  |  | 1656 (61.8) | 1022 (38.2) |  |  | 515 (50.0) | 514 (50.0) |  |
| ＞21 | 773 (77.0) | 231 (23.0) |  |  | 190 (59.9) | 127 (40.1) |  |  | 79 (61.7) | 49 (38.3) |  |
| AST/ALT ratio |  |  | 0.028 |  |  |  | 0.03 |  |  |  | 0.454 |
| 0.8-1.5 | 3436 (70.1) | 1465 (29.9) |  |  | 845 (59.5) | 575 (40.5) |  |  | 272 (49.7) | 275 (50.3) |  |
| ＜0.8 | 827 (70.3) | 349 (29.7) |  |  | 216 (60.5) | 141 (39.5) |  |  | 64 (50.0) | 64 (50.0) |  |
| ＞1.5 | 3195 (72.5) | 1209 (27.5) |  |  | 785 (64.4) | 433 (35.6) |  |  | 258 (53.5) | 224 (46.5) |  |
| Ccr (mol/min) |  |  | 0.256 |  |  |  | 0.361* |  |  |  | 0.963 |
| 72-172 | 1605 (71.6) | 636 (28.4) |  |  | 357 (64.1) | 200 (35.9) |  |  | 112 (50.2) | 111 (49.8) |  |
| ＜72 | 1788 (69.5) | 786 (30.5) |  |  | 450 (61.1) | 286 (38.9) |  |  | 127 (50.4) | 125 (49.6) |  |
| ＞172 | 8 (72.7) | 3 (27.3) |  |  | 2 (100.0) | 0 (0.0) |  |  | 0 (0.0) | 1 (100.0) |  |
| WBC (10^9^/L) |  |  | 0.002 |  |  |  | 0.006 |  |  |  | 0.003 |
| 3.5-9.5 | 5173 (69.8) | 2235 (30.2) |  |  | 1310 (61.0) | 838 (39.0) |  |  | 415 (51.4) | 393 (48.6) |  |
| ＜3.5 | 1136 (71.3) | 457 (28.7) |  |  | 285 (57.6) | 210 (42.4) |  |  | 79 (41.4) | 112 (58.6) |  |
| ＞9.5 | 1219 (74.1) | 425 (25.9) |  |  | 315 (67.3) | 153 (32.7) |  |  | 116 (58.6) | 82 (41.4) |  |
| NEUT (10^9^/L) |  |  | 0.008 |  |  |  | <0.001 |  |  |  | <0.001 |
| 40-75 | 5006 (70.2) | 2126 (29.8) |  |  | 1232 (61.2) | 780 (38.8) |  |  | 398 (51.8) | 370 (48.2) |  |
| ＜40 | 935 (69.3) | 415 (30.7) |  |  | 260 (54.5) | 217 (45.5) |  |  | 71 (38.2) | 115 (61.8) |  |
| ＞75 | 1587 (73.4) | 576 (26.6) |  |  | 418 (67.2) | 204 (32.8) |  |  | 141 (58.0) | 102 (42.0) |  |
