# Supplementary material for: Characteristics and risk factors for readmission in HIV-infected patients with Talaromyces marneffei infection
Source: PLoS Negl Trop Dis. 2023 Oct 10;17(10):e0011622. doi: 10.1371/journal.pntd.0011622 (PMC10564132; doi:10.1371/journal.pntd.0011622)
Supplement: S6 Table — (DOCX) [file pntd.0011622.s006.docx]

**S6 Table. Basic demographic information and ART status among the *T. marneffei* infection population at first admission before and after 1:1 propensity score matching**

|  | Before adjustment | | | After adjustment | | |
| --- | --- | --- | --- | --- | --- | --- |
| Variable | No (n=1453) | Yes (n=288) | *p* | No (n=288) | Yes (n=288) | *p* |
| Age |  |  | 0.019 |  |  | 0.941 |
| <20 | 32 (2.2) | 8 (2.8) |  | 9 (3.1) | 8 (2.8) |  |
| 20-40 | 638 (43.9) | 151 (52.4) |  | 153 (53.1) | 151 (52.4) |  |
| 41-60 | 576 (39.6) | 103 (35.8) |  | 97 (33.7) | 103 (35.8) |  |
| >60 | 207 (14.2) | 26 (9.0) |  | 29 (10.1) | 26 (9.0) |  |
| Sex |  |  | 0.498 |  |  | 0.83 |
| Male | 1202 (82.7) | 233 (80.9) |  | 236 (81.9) | 233 (80.9) |  |
| Female | 251 (17.3) | 55 (19.1) |  | 52 (18.1) | 55 (19.1) |  |
| Nationality |  |  | 0.019 |  |  | 1.000* |
| Han | 884 (60.8) | 158 (54.9) |  | 159 (55.2) | 158 (54.9) |  |
| Zhuang | 529 (36.4) | 127 (44.1) |  | 127 (44.1) | 127 (44.1) |  |
| Other | 40 (2.8) | 3 (1.0) |  | 2 (0.7) | 3 (1.0) |  |
| Marital status |  |  | 0.022 |  |  | 0.287* |
| Single, divorced or widowed | 570 (39.2) | 102 (35.4) |  | 107 (37.2) | 102 (35.4) |  |
| Married | 830 (57.1) | 183 (63.5) |  | 181 (62.8) | 183 (63.5) |  |
| Other | 53 (3.6) | 3 (1.0) |  | 0 (0.0) | 3 (1.0) |  |
| Occupation |  |  | 0.988 |  |  | 0.936 |
| Farmer | 789 (54.3) | 156 (54.2) |  | 159 (55.2) | 156 (54.2) |  |
| Unemployed, retired | 296 (20.4) | 60 (20.8) |  | 61 (21.2) | 60 (20.8) |  |
| Other | 368 (25.3) | 72 (25.0) |  | 68 (23.6) | 72 (25.0) |  |
| ART |  |  | 0.484 |  |  | 0.723 |
| No | 1124 (77.4) | 217 (75.3) |  | 220 (76.4) | 217 (75.3) |  |
| Yes | 208 (14.3) | 49 (17.0) |  | 51 (17.7) | 49 (17.0) |  |
| Unknow | 121 (8.3) | 22 (7.6) |  | 17 (5.9) | 22 (7.6) |  |
